# Two cases of transplant-acquired food allergy who developed resensitization after a negative oral food challenge

**DOI:** 10.1186/s13223-023-00784-5

**Published:** 2023-03-22

**Authors:** Akiko Nakaoka, Takayasu Nomura, Kazuyoshi Ozeki, Tomotaka Suzuki, Shigeru Kusumoto, Shinsuke Iida, Shinji Saitoh

**Affiliations:** 1 Department of Pediatrics and Neonatology, Graduate School of Medical Sciences, Nagoya City University, 1 Kawasumi, Mizuho, Nagoya, Aichi 467-8601 Japan; 2 Department of Hematology and Oncology, Graduate School of Medical Sciences, Nagoya City University, Nagoya, Japan

**Keywords:** Cord blood transplantation, Liver transplantation, Oral food challenge, Resensitization, Transplant-acquired food allergy

## Abstract

**Background:**

Cases of food allergy after hematopoietic stem cell and solid organ transplantation in previously nonallergic transplant recipients were reported as transplant-acquired food allergy (TAFA), but information about its long-term outcome is still limited. A phenomenon where patients reacquire food allergy by resuming daily consumption after a negative oral food challenge has not yet been reported.

**Case presentation:**

We report two cases of TAFA after liver transplantation and cord blood transplantation. In each case, the threshold of daily consumption to cause allergic symptoms decreased when a negative oral food challenge was obtained.

**Conclusions:**

Our cases show an importance of gastrointestinal tract as a route of food sensitization because thresholds that caused allergic reactions decreased during their resuming process. We need to be careful with possible resensitization once a negative substantial dose was confirmed.

## Background

A common mechanism of food allergy (FA) is the breakdown of immunologic and clinical tolerance to an ingested food, but its detailed mechanism is still unclear [1]. Cases of FA after hematopoietic stem cell and solid organ transplantation in previously nonallergic transplant recipients were reported as transplant-acquired food allergy (TAFA) [2], but information about its long-term outcome is still limited [3–6]. A phenomenon where patients reacquire FA by resuming daily consumption after a negative oral food challenge (OFC) has not yet been reported. Because the mechanisms of TAFA may help understand general FA, unique cases of TAFA with long-term outcomes should be accumulated. Here, we report two cases of TAFA after liver transplantation and cord blood transplantation (CBT). In each case, the threshold of daily consumption to cause allergic symptoms decreased when a negative OFC was obtained.

## Case presentation

### Case 1

A 1-year-and-10-month-old boy who was diagnosed with Alagille syndrome received a live-donor liver transplant from his nonallergic father. His food allergy was diagnosed by immunoglobulin E (IgE) associated immediate symptoms related to food ingestion, such as vomiting with wheat, and vomiting and systemic urticaria with soybean (Table 1). No allergic history except for the food allergy had been reported at the transplantation. His transplantation was uneventful, and tacrolimus has been used as a preventive agent for rejection. Egg allergy, which is the most common food allergy in this age group in Japan, was suspected because he experienced allergic reactions to wheat and soybean; he also had sensitization to hen’s egg white (EW) (Table 1). As he had avoided to eat hen’s egg completely, initial consumption of EW was planned in our hospital when he was 2 years and 5 months old. Consequently, the OFC was negative with 15 g of boiled EW, and he was initiated on daily consumption of boiled EW by adding stepwise doses (1 g every 3 days). The consecutive daily consumption was unremarkable until he encountered systemic urticaria with 26 g of boiled EW about 1 month after the negative OFC. His systemic urticaria was reproducible with lower doses of boiled EW for a few days without contamination of wheat and soybean, and respiratory symptoms including wheezing from consuming 9 g of boiled EW finally gave him up to continue eating. High titers of EW-specific IgE were accompanied by allergic reactions, and the titers gradually decreased with the complete elimination of hen’s egg consumption (Table 1). Regarding wheat and soybean, daily consumption without any concern has been accomplished in his natural course of food allergy.

**Table 1:** Series of food-specific IgE in Case 1

Age	8 m	1y8m	2y2m	2y4m	2y8m	3y2m	4y2m
Events		Liver transplantation		OFC^a^			
Total IgE, IU/mL	121		186		1300	2490	284
Specific IgE (CAP^b^)							
Egg white, U_A_/mL	8.19		6.86		40.5	15.7	5.33
Ovomucoid, U_A_/mL	< 0.34		0.26		26.1	8.44	1.93
Wheat, U_A_/mL	6.96		1.56		8.82	14.1	2.52
Soybean, U_A_/mL	7.69		1.32		9.63	11.9	1.46

^a^OFC, oral food challenge

^b^CAP, capsulated hydrophilic carrier polymer

### Case 2

A 51-year-old male without any history of allergic disease was diagnosed with anaplastic large cell lymphoma (ALCL), and he received cyclophosphamide, doxorubicin, vincristine, and prednisone (CHOP) chemotherapy as an induction regimen. Although salvage-intensive treatment, including a CBT, was required for CHOP-refractory ALCL, the CBT had achieved a complete response. Unfortunately, despite the prophylactic use of tacrolimus, grade 3 intestinal graft-versus-host disease (GVHD) characterized by vomiting and watery diarrhea (> 1500 mL per day) was diagnosed based on histological GVHD findings 2 months after. Refractory diarrhea forced him to consume ingredient nutrition with small amounts of snacks. GVHD therapy using systemic steroids and mesenchymal stem cell therapy was effective.

Six months after the CBT, he was finally allowed to consume solid food. On the next day, he unexpectedly experienced fever, frequent vomiting, diarrhea, and refractory hypotension with unknown mechanism that required continuous noradrenaline injection. Many kinds of food antigen could be contaminated in the solid foods because no attention had been paid for his possible food allergy. But Baumkuchen, that is a desert containing egg, milk, and wheat, and yogurt were critical to cause immediate severe hypotension in the episode. Blood examination revealed that he was sensitized to multiple antigens (Table 2), including hen’s egg, milk, and wheat. After the diagnosis of FA, he never experienced allergic reactions by avoiding these diets. OFC was conducted after 1 year and 9 months of the CBT, and his negative allergic status was proven through the boiled egg challenge with one whole egg. Daily consumption of one whole egg was started without any allergic symptoms, but it finally caused vomiting and watery diarrhea on the seventh day. The symptoms were reproducible with the next boiled egg challenge with one whole egg after 1 week of the first episode, and resensitization to EW supported his allergic reaction to it (Table 2). Although specific IgE assays (i.e., MAST and CAP assay) were used for the assessment, because MAST assay is useful for screening and CAP assay is quantitative for management of diagnosed food allergy, his sensitization was obvious in the same assay [8]. Limited information related to food allergy of the donor was available in a CBT setting.

**Table 2:** Series of food-specific IgE in Case 2

Age	51y11m	52y5m	52y8m	52y11m	53y4m	53y5m	53y8m	53y10m	54y0m
Events	CBT^a^						OFC^b^		
Total IgE, IU/mL		185	N.D	N.D	N.D	25.9		156	53.6
Specific IgE (MAST^c^)									
Egg white, LC^d^		1.11	2.76	2.29	12.6	N.D		N.D	N.D
Milk, LC		0.77	3.56	5.09	1.01	N.D		N.D	N.D
Wheat, LC		4.84	24.0	4.30	0.84	N.D		N.D	N.D
Specific IgE (CAP^e^)									
Egg white, U_A_/mL		N.D	N.D	N.D	N.D	< 0.10		1.60	0.71
Ovomucoid, U_A_/mL		N.D	N.D	N.D	N.D	< 0.10		3.29	0.90
Milk, U_A_/mL		N.D	N.D	N.D	N.D	< 0.10		0.14	0.26
Wheat, U_A_/mL		N.D	N.D	N.D	N.D	< 0.10		< 0.10	< 0.10

*N.D.* Not determined

^a^CBT, cord blood transplantation

^b^OFC, oral food challenge

^c^MAST, multiple antigen simultaneous test

^d^LC, lumi count

^e^CAP, capsulated hydrophilic carrier polymer

## Discussion and conclusions

Although these cases had different backgrounds in terms of age and type of organ or hematopoietic transplantation, both gave up consuming an allergen, which was proven to be negative in the OFC. In Case 1, a gradual decrease in thresholds that caused allergic reactions was observed within a month of resuming hen’s egg intake. This might not be a case of TAFA because he never consumed it when the liver transplant was done. Further discussion is needed to diagnose TAFA for such cases. Ovomucoid-specific IgE was reported as a useful marker of symptomatic egg allergy [7]. Although pre transplant clinical response was unclear in this case, it might be a clinical related marker of TAFA because the enhancement was obvious with daily consumption of boiled egg. Case 2 was a rare case of TAFA after CBT, and only 16 cases were summarized in a recent case report [6]. TAFA is transient in most pediatric cases after CBT, but less is known in adult cases. We need to be careful with possible resensitization once a negative substantial dose was confirmed in an OFC.

Some mechanisms of TAFA are proposed in clinical and animal studies [2]. A passive transfer of donor immune cells is the most frequently proposed mechanism. Allergen-specific IgE, allergen-specific lymphocytes, liver-resident dendritic cells, and sinusoidal endothelial cells were reported as sources of immune cells. Other possible mechanisms are the action of tacrolimus [9] and immature gastrointestinal and immune system [10]. Tacrolimus promotes Th2 responses to induce IgE secretion from B cells and increases intestinal permeability [9]. Our cases of TAFA are surprising because they were sensitized by an ingested food for a relatively limited period. Although the mechanism of sensitization has been recently focused on epicutaneous route [11], these cases encouraged us to refocus on the role of the gastrointestinal tract.

We experienced two cases of TAFA. Because thresholds that caused allergic reactions decreased during their resuming process, the cases show an importance of gastrointestinal tract as a route of food sensitization. Unique cases should still be accumulated to clarify the detailed mechanism of TAFA; this might shed light on the origin of FA.

## Data Availability

Not applicable.

## Abbreviations

ALCL: Anaplastic large cell lymphoma
CAP: Capsulated hydrophilic carrier polymer
CBT: Cord blood transplantation
EW: Egg white
FA: Food allergy
GVHD: Graft-versus-host disease
Ig: Immunoglobulin
LC: Lumi count
MAST: Multiple antigen simultaneous test
OFC: Oral food challenge
TAFA: Transplant-acquired food allergy
